# The Relationship between Environmental Regulation, Pollution and Corporate Environmental Responsibility

**DOI:** 10.3390/ijerph18158018

**Published:** 2021-07-29

**Authors:** Mengxin Wang, Gaoke Liao, Yanling Li

**Affiliations:** 1 Guangzhou Institute of International Finance, Guangzhou University, Guangzhou 510006, China; wangmx2016@163.com; 2 School of Economics and Statistics, Guangzhou University, Guangzhou 510006, China; 2112064100@e.gzhu.edu.cn

**Keywords:** environmental regulation, environmental pollution, corporate environmental responsibility

## Abstract

The rapid economic development has severely damaged the ecological environment and affected public health. Firms are the main source of pollution; thus, corporate environmental responsibility (CER) has attracted great attention from the government, shareholders and the public. This study used both the fixed effects model and the system GMM (Generalized Method of Moments) model to examine the relationship between environmental pollution, environmental regulations and CER for 30 provinces in China, over the period 2005 to 2015. This study drew the following results: first, mandatory CER disclosure policy can significantly decrease environmental pollution. Second, an inverted U-shaped relationship exists between environmental regulations and environmental pollution. Third, environmental pollution has a positive impact on CER. Fourth, an inverted U-shaped relationship exists between environmental regulations and CER. Therefore, it is necessary to find a balance between environmental regulations affecting environmental pollution and CER so that they can effectively reduce environmental pollution and increase the enthusiasm of firms to carry out environmental responsibility activities.

## 1. Introduction

China’s economic development has made remarkable achievements in the world, but the environmental pollution caused by rapid economic development has severely damaged the ecological environment and affected public health [1,2]. This is based on concerns about their own lives and health. On the one hand, the public requires the government to introduce various measures to protect the environment [3]. On the other hand, the public requires firms to assume corporate environmental responsibilities (CER) and reduce the damage to the ecological environment caused by production and business activities [4]. As the main consumers of micro-economic entities and resources and energy, firms are not only the main producers of environmental pollution, but also the main undertakers of environmental protection. The environmental responsibility of firms has attracted great attention from the government, shareholders and the public [5,6]. In this context, it is of great significance to explore the relationship between environmental pollution, government environmental regulations and corporate environmental responsibility.

In order to solve the continuous serious pollution problem, the Chinese government has strengthened environmental regulations to prevent and solve the problems of environmental pollution and overcapacity [7,8]. It is generally believed that environmental regulation refers to the relevant policies and measures of the government to reduce pollution and promote green production by restricting the production and operation activities of firms. Currently recognized environmental regulations include government-mandatory environmental regulations that directly control the resource utilization behavior of firms through non-market means and market-incentive environmental regulations that use market means to provide indirect incentives, such as tax relief and technical support, to firms.

Environmental regulations are the government’s means of controlling the use of environmental resources and are of great significance to strengthening environmental protection [9,10]. However, can China’s environmental regulations effectively improve the environment and increase the CER behavior of firms? Wang et al. [11] noted that China’s total investment in the treatment of environmental pollution has been increasing; however, the discharge of major pollutants is still increasing. The firms, especially in heavy polluting industries, are the main source of pollution [12]. Firms that take CER can control pollution emissions from the source. If it can be verified that provincial environmental regulations have a positive effect on CER, not only can it provide new ideas for improving CER from a macro level, but it can also curb environmental pollution.

At present, most of the relevant research on CER is based on a micro perspective, mainly examining the relationship between CER and corporate environmental performance and economic performance. Most of the relevant research on environmental regulation is based on a macro perspective, mainly examining the relationship between environmental regulation and sustainable development. We extend the literature by empirically analyzing the effects of environmental regulation on environmental pollution and CER, and the effects of environmental pollution on CER. The first contribution of this paper is to study CER from the perspective of environmental pollution, while other studies have mainly studied CER from the corporate characteristics. Then, this paper discovers the non-linear impact of environmental regulations on environmental pollution and CER. Therefore, this paper provides marginal contribution for CER development theory and practice.

The rest of the paper is organized as follows: Section 2 lays out the brief literature review and research hypothesis. Section 3 introduces the data and methodology for empirical research, and measures CER and environmental regulations of each province. In Section 4, we analyze the relationship between environmental regulation, environmental pollution and CER. Section 5 provides the main conclusions.

## 2. Literature Review and Research Hypothesis

### 2.1. Environmental Regulation and Environmental Pollution

In terms of research on the relationship between environmental regulation and pollution, the current hypotheses are mainly based on the hypothesis of forced emission reduction and the green paradox. Scholars who support the first hypothesis believe that the purpose of the government’s implementation of environmental regulations is to reduce pollution emissions. Specifically, on the one hand, the government levies energy taxes on energy producers and users and levies pollution discharge fees on polluters, which increases the production costs and environmental costs of firms, thereby helping to reduce energy consumption and pollution emissions [13,14]. On the other hand, the government encourages firms to use alternative energy by subsidizing clean energy, which will also reduce the consumption of fossil energy [15]. Neves et al. [16] studied the effects of market-based regulations and regulatory policies on carbon dioxide emissions, and they found that environmental regulation is effective in cutting CO2 emissions in the long-run. Zhang et al. [17] showed that China’s current environmental regulation had effectively inhibited the haze pollution and achieved the expected effects.

Scholars who support the green paradox view believe that the environmental regulations will accelerate pollution emissions. With the increase in the intensity of environmental regulations, energy producers will accelerate the progress of mining, hoping to sell out energy assets before the implementation of the new environmental standards, which will accelerate energy consumption and lead to a rapid expansion of pollution emissions [18]. Hao et al. [19] indicate that current environmental control measures and regulations of China have not achieved the desired goal of controlling and reducing pollution. Zhang et al. [20] indicate that the Chinese-style fiscal decentralization makes the environmental policy significantly promote carbon emissions, leading to a green paradox. The relationship between environmental regulation and environmental pollution has not yet reached a consistent conclusion, which may be due to the non-linear relationship between environmental regulation and environmental pollution. Thus, this paper proposes:

**Hypothesis** **1** **(H1).** *Environmental regulation has a U-shaped effect on the environmental pollution.*

### 2.2. Environmental Regulation and Corporate Environmental Responsibility

The research results on the effect of environmental regulations on CER can be roughly divided into two viewpoints: the inhibition view and the promotion view.

The inhibition view believes that environmental regulations will weaken the competitiveness of firms, and firms will therefore reject environmental responsibility. On the one hand, environmental regulations force firms to control environmental pollution through laws and regulations. While complying with environmental regulations, firms need to invest in research and development of pollution control technologies, purchase pollution control equipment, and apply for pollution discharge permits. These expenditures force firms to internalize external costs. It will affect the operating efficiency of the firm and weaken the market competitiveness of the firm [21,22,23]. In addition, in order to comply with environmental regulations, firms also need to change the existing process and production technology, which will bring the risk of reduced production efficiency to the firm [24]. On the other hand, strict local environmental regulations that may affect local firms’ Economic benefits will turn away potential investors [25]. These investors may choose to invest in areas with relatively loose environmental regulations. When firms are forced to internalize environmental management costs, they have to embezzle part of the funds used for production and operation to invest in environmental pollution. This will have a crowding out effect on productive investment and increase their own operating risks [26]. Therefore, the inhibition view believes that in the face of environmental regulations, firms are easily caught in a dilemma between economic benefits and environmental responsibility, and some firms may even resist taking environmental responsibility.

The promotion view believes that while compelling firms to assume environmental responsibilities, environmental regulations will stimulate their innovation capabilities, thereby improving production efficiency and enhancing their competitiveness [27]. On the one hand, under the pressure of environmental regulations, firms will increase R&D efforts to enhance their own innovation capabilities, save costs and create more economic benefits by improving energy efficiency and reducing waste generation rates. Although environmental regulations will have a crowding-out effect on the productive investment of firms, under the constraints of environmental regulations, firms can improve the original production mode to maximize resource utilization [28,29]. Therefore, in the long run, the benefits of environmental regulations for firms will make up for the losses caused by the internalization of environmental costs. On the other hand, with the spread of environmental awareness worldwide, investors will not only benefit from economic benefits, but also pay attention to the impact of corporate production activities on the environment. Compared with those firms that do not comply with environmental regulations and do not assume environmental responsibilities, firms that have a green operating system and actively assume environmental responsibilities will be favored by investors because they have less risk of environmental penalties [30]. In addition, under the pressure of environmental regulations, firms will proactively disclose environmental information in an open and transparent manner, which helps establish the firm’s communication and trust with upstream and downstream suppliers and investors to gain green product competitiveness [31]. The relationship between environmental regulation and CER has not yet reached a consistent conclusion, which may be due to the non-linear relationship between environmental regulation and CER. Thus, this paper proposes:

**Hypothesis** **2** **(H2).** *Environmental regulation has a U-shaped effect on the CER.*

### 2.3. Corporate Environmental Responsibility and Environmental Pollution

Based on different CER motivations, CER can be divided into mandatory disclosure and voluntary disclosure [32]. In order to cope with the impact of external pressure, firms will be forced to make mandatory environmental information disclosure. This kind of external pressure includes one type, which is the pressure imposed by the government on the enterprise by the administrative order or the laws and regulations. This is the direct compulsory pressure, and the pressure must be dealt with. Its influence is great and direct. The other type is pressure from outside social public opinion and moral condemnation, which is an indirect spiritual soft binding force [33,34]. Chen et al. [35] found that mandatory disclosure alters firm behavior and decreases industrial effluents and SO_2_ emission levels. Chen et al. [36] found that mandatory disclosure exerts a significantly positive impact on residents’ sense of happiness by reducing air pollution. Therefore, mandatory disclosure will inhibit environmental pollution.

Compared with mandatory disclosure, firms that voluntarily disclose CER information pay more attention to the interests of stakeholders [37,38]. This is because voluntary disclosure is regarded as a voluntary exchange of information shared between companies and other stakeholders based on economic interests, and it lacks uniform restrictions and regulations. Therefore, voluntary disclosure of CER participation is highly subjective and random. Voluntary disclosure can convey information about the firm’s core capabilities and show its own advantages [39,40,41]. It is widely recognized that environmental pollution directly and indirectly affects public health and firm production. Pope III et al. [42] found that the decrease of cubic meters in the concentration of fine particulate matter was associated with an estimated increase in mean life expectancy. Chang et al. [43] found that higher levels of air pollution decrease worker productivity. In the context of voluntary disclosure, environmental pollution will prompt firms to carry out more CER activities.

In summary, this paper proposes the following hypotheses:

**Hypothesis** **3** **(H3).** *Mandatory CER disclosure policy can inhibit the level of environmental pollution.*

**Hypothesis** **4** **(H4).** *The more severe the environmental pollution, the more likely firms would be to exercise better CER.*

## 3. Sample, Variables, and Methodology

### 3.1. Sample

In order to examine the effect of environmental regulation and pollution on CERD, this paper selected 30 provinces in mainland China from 2005 to 2015 for research. Considering the integrity of the data, the sample excluded Tibet.

In order to study the CERD situation in different provinces, this paper used the average situation of listed firms in each region to reflect the provincial CERD level. Therefore, we selected A-share listed firms in China’s Shanghai and Shenzhen stock markets from 2008 to 2015 to measure CERD. The data sources used were the CSMAR database, CNRDS database and WIND database. This paper processed the original data according to the following standards: (1) Exclude the samples of firms with severe financial data missing; (2) Exclude the samples of ST and * ST firms, because these firms are at risk of being delisted, and the information they disclose is very high. They may not reflect the true financial status; (3) Exclude the sample of listed firms in the year of IPO, because the firm may have earnings manipulation at this time; (4) Exclude firms that have not issued a standard unqualified opinion by the accounting firm, because these firms are very likely to have major financial problems. After the above screening, the final sample contained a total of 524 listed firms with a total of 4192 panel data from 30 provinces in China. Table 1 indicates the geographical distribution of sample firms in this study.

### 3.2. Variables

#### 3.2.1. Environmental Pollution

Considering that industrial pollution is the most important source of environmental pollution, while taking into account the availability of data, this paper selected sulfur dioxide emissions per square kilometer (SO_2_), industrial fumes emissions per square kilometer (SMOKE) and industrial effluent emissions per square kilometer (WATER) to measure the environmental pollution level of each region.

#### 3.2.2. Corporate Environmental Responsibility

The environmental information report disclosed by the firm is the main way for investors to understand the environmental responsibility of the firm. Most of the research has been based on the environmental information disclosed by the firm to measure the environmental responsibility of the firm. This paper drew on the research methods of Li et al. [44], and it designed a total of 13 specific indicators to measure corporate environmental responsibility from five dimensions: legal consciousness, social rating, process environmental protection, low-carbon technology and green management.

The first was legal consciousness. This reflects a firm’s strengthening of legal awareness and compliance with environmental-related laws and regulations in the production and operation process, measured by the three indicators of whether the firm refers to the GRI (Global Reporting Initiative) Sustainability Reporting Guidelines, whether it discloses the environment and sustainable development, and whether it has received environmental penalties. We referred to the GRI Sustainability Reporting Guidelines to prove that the firm was in line with the development direction of green production. Disclosure of the environment and sustainable development and whether it had received environmental penalties reflects whether the firm complies with environmental protection laws and regulations to support environmental protection related policies.

The second was social rating. Social rating was to examine whether a firm had a good social reputation in terms of the environment. A good social reputation will bring a higher degree of market recognition to the firm. Social rating refers to the evaluation of a firm’s environmental management system made by a third-party organization or society, including environmental recognition and other environmental advantages.

The third was process environmental protection. It was to measure the environmental impact of firms in the production and operation process, including two indicators of circular economy and measures to reduce three wastes. Firms that adopt a circular economy have stronger environmental responsibilities, and the use of measures to reduce the three wastes is an important manifestation of the development of green production by firms.

The fourth was low-carbon technology. This reflects the use of green production technologies by firms, including two indicators of energy-saving and environmentally beneficial products.

The fifth was green management. It measured whether the firm included green environmental protection into the planning scope in the management and operation process, including whether it was verified by a third-party organization, CSR (corporate social responsibility) vision, environmental certification and green office. Information reports verified by third-party organizations are more objective. Firms that have passed ISO14001 certification for their environmental management system and firms that have green office policies or measures and CSR vision have stronger environmental responsibilities.

The environmental responsibility measurement indicator system established in this paper is shown in Table 2. In order to keep the direction of all indicators consistent, firms that receive environmental penalties had a score of 0, and those that had not received environmental penalties had a score of 1. In order to avoid the subjectivity of weighting indicators, this paper assigned all indicators the same weight. The score of each dimension was the sum of the scores of each indicator, and the total score of environmental responsibility was the sum of the scores of each dimension.

#### 3.2.3. Environmental Regulation

Government-compulsory environmental regulation refers to the government’s strict environmental standards, through the implementation of compulsory measures such as transformation and shutdown of firms, to limit the pollution discharge of firms in the process of production and operation. The main body of government-mandatory environmental regulations is the government. The government formulates environmental protection regulations, and firms are forced to implement these regulations under the supervision of the government and society. This type of environmental regulation usually has a good short-term effect, but due to poor flexibility and high cost, firms cannot fundamentally respond to environmental regulations by improving resource utilization and reducing the rate of pollutants.

Market-incentive environmental regulation means that the government uses market-oriented means such as taxation, subsidies and credit to enable firms to take the initiative to reduce environmental pollution through a series of measures such as energy conservation and emission reduction. The main body of market-incentive environmental regulation is firms. The government mainly encourages firms to actively carry out pollution prevention and control and technological upgrading through policies such as technology subsidies, pollution control subsidies and tax reductions. Compared with government-mandatory environmental regulations, market incentives environmental regulations are more flexible, and firms have more initiative, but at the same time, there are problems such as lagging effects and higher uncertainty.

At present, the academic community has not yet reached a unified conclusion on the selection of indicators. With reference to the indicators used in most documents and based on the availability of data for government-mandatory environmental regulations, this paper selected the proportion of industrial pollution control investment in industrial added value; the total investment in environmental governance and the comprehensive utilization rate of industrial solid waste are measured by three indicators [45,46]. For market-incentive environmental regulations, this article used provincial pollution discharge fees as the measurement indicators.

Through these four indicators, this article constructed a provincial environmental regulation measurement system to comprehensively examine the intensity of environmental regulation in each province. The processing methods for indicator data were as follows:

First, we standardized the indicator data. In order to eliminate the influence of different measurement units and calibers of different indicators, the indicator data was processed in a dimensionless manner. Then, we assigned a weight coefficient to the indicator. In order to eliminate subjectivity, this paper adopted an entropy weight method to weight the indicators.

#### 3.2.4. Control Variables

Different provinces in China are quite different in terms of geographic location, population, resource storage and economic development, which may lead to large differences in environmental regulations in different provinces. With reference to relevant literature, this article selected a total of six control variables at the micro-level and macro-level, among which the micro-level control variables were the natural logarithm of total assets (SIZE), the total debt divided by total assets (DEBT) and return on assets (ROA); macro-level control variables were gross domestic product growth (GDP), industrial added value growth (IND) and urban population divided by total population (CITY) [47,48].

Table 3 presents the descriptive statistics for all variables in the empirical study. The maximum value of CER was 7.2119, the minimum value was 1 and the average was 4.3682. This suggests that the overall quality of CER was still at a relatively low level in China. Moreover, we find that the level of environmental pollution varies significantly in different provinces.

### 3.3. Methodology

This paper constructed Model (1) to examine the effect of the comprehensive index of provincial environmental regulation and CER Policy on environmental pollution.

Pollution_it_ = β_0_ + β_1_DCER_it_ + β_2_ER_it_ + β_3_ER^2^_it_ + ∑aX_it_ + ε_it_ (1)

where i is various provinces, and t is time. Pollution presents SO_2_, SMOKE and WATER, and X is the control variable, and β_0_ is intercept, while, DCER represents the mandatory environmental disclosure policy; the DCER value before 2008 is 0, and after 2008, it is 1. ER represents the environmental regulation; ER^2^ the square of environmental regulation. ε is white noise error disturbance.

Then, we constructed Model (2) to examine the effect of environmental regulation and Pollution on CER.

CER_it_ = β_0_ + β_1_DCER_it_ + β_2_ER_it_ + β_3_ER^2^_it_ + ∑aX_it_ + ε_it_ (2)

Considering that CER may be affected by its previous environmental responsibility, this paper further established a dynamic panel model to study the impact of environmental regulation and Pollution on CER. The model can be expressed by Equation (3).

CER_it_ = β_0_ + β_1_CER_it−1_ + β_2_DCER_it_ + β_3_ER_it_ + β_4_ER^2^_it_ + ∑aX_it_ + ε_it_ (3)

## 4. Results

To avoid spurious regression, we tested the stationarity of all variables used in this study. We used the Levin–Lin–Chu (LLC) and the Fisher-Augmented Dickey–Fuller (Fisher-ADF) method to perform the panel unit root test. When the *p*-value was less than 0.01, we could conclude that the variable was stationary. Table 4 shows the statistics and corresponding *p*-values. We can see that only SO_2_ failed the LLC and ADF test, and other variables passed the stationarity test. Therefore, we examined the stationarity of the first-order difference of SO_2_. In Table 4, we can see the first-order difference of SO_2_ was a stationary process, which shows that our data were stationary and suitable for the next step of analysis.

Table 5 shows the regression result of the environmental regulation and CER on environmental pollution. The regression analysis in Table 5 was based on the results of the fixed effect model from 2005 to 2015. In order to study whether the CER policy will affect environmental pollution, this paper used the mandatory disclosure of CER-related information required by the Chinese government in 2008 as the policy implementation node to set the dummy variable DCER. The DCER value before 2008 was 0, and after 2008, it was 1. From Table 5, we could find that whether it was SO_2_, SMOKE or WATER as an indicator of environmental pollution, the coefficient of DCER was remarkably negative at the significance level of 1%. This indicates that the mandatory CER disclosure policy could significantly decrease the CER environmental pollution. This result verifies H3.

The relationship between environmental regulation and environmental pollution was nonlinear. From Table 5, we could find that when SO_2_ was used as the dependent variable, the coefficient of environmental regulation was significantly positive at 10%, and the coefficient of the square term was remarkably negative at the significance level of 10%. This shows that the inverted U-shaped relationship exists between environmental regulation and SO_2_. In the early stages of environmental regulation, the increase in level of environmental regulation will not reduce SO_2_. Only when environmental regulation reaches a certain level, environmental regulations can help reduce SO_2_. When SMOKE was used as the dependent variable, environmental regulation showed an insignificant impact on CER. When WATER was used as the dependent variable, the coefficient of environmental regulation was significantly positive at 5%, and the coefficient of the square term was negative at the significance level of 5%. This shows that the inverted U-shaped relationship exists between environmental regulation and WATER. Therefore, the impact of environmental regulations on different pollutants is heterogeneous. The results verify H1.

This paper used both the fixed effects model and the system GMM model to analyze the impact of environmental pollution and environmental regulations on CER from 2008 to 2015. Table 6 shows the regression result of the environmental regulation and environmental pollution on CER.

From the regression results of the fixed effect model, we could find that the coefficient of SO_2_ was 0.994, and the coefficient of SMOKE was 0.0823; both of them were significantly positive at 1%. However, the WATER showed an insignificant impact on CER. This shows that different environmental pollution will have different effects on CER. SO_2_ and SMOKE are the main air pollutants, and the increase in air pollution has prompted firms to increase their environmental responsibility. This result verifies H4. However, water pollution did not have a significant impact on CER. This coincides with the background that haze pollution has led government departments to strengthen air pollution control.

When SO_2_, SMOKE and WATER were used as independent variables. The coefficients of environmental regulation were all remarkably positive at the significance level of 1%, and the coefficient of the square term were all negative at the significance level of 5%. This indicates that the inverted U-shaped relationship between environmental regulation and CER was robust. In the process of continuous strengthening of environmental regulations, the increase in level of environmental regulation will promote CER. When environmental regulation reaches a certain level, excessive environmental regulations will lead to a decline in the effect of promoting CER. The results verify H2.

From the regression results of the system GMM model, in all the regression results, the moment estimation passed the Sargan test, indicating that the choice of instrumental variables was effective. From the *p* values of AR (1) and AR (2), the random disturbance term had a first-order serial correlation and no second-order serial correlation, which conformed to the assumption of the validity of the system GMM method. The impact of the first-order lag term of CER on the current CER was positive at the significance level of 1%, which indicates that CER has a dynamic and positive continuity characteristic.

Environmental pollution has a positive impact on CER. The coefficient of SO_2_ and the coefficient of the SMOKE were significantly positive, which indicates that SO_2_ and SMOKE pollution promote more environmentally responsible behaviors of firms. Air pollution is the main factor driving CER, and water pollution does not affect CER. This result also verifies H4. When SO_2_ was used as an independent variable, the inverted U-shaped relationship still existed between environmental regulation and CER. However, when SMOKE and WATER were used as independent variables, the coefficient of environmental regulation was significantly positive at 1%, but the square term of environmental regulation coefficient failed the significance test. This shows that environmental regulations will promote firms to carry out more CER activities. The results also verify H2.

## 5. Conclusions

In this paper, we use the fixed effects model and the system GMM model to examine the relationship between environmental pollution, environmental regulations and CER for 30 provinces from China, over the period of 2005 to 2015. First, we separately measured the core variables of this paper, environmental regulation and CER, and used sulfur dioxide emissions, industrial fumes emissions and industrial effluents emissions as environmental pollution indicators. Then, we studied the impact of environmental regulations and mandatory environmental disclosure policies on different pollutants. Finally, we analyzed the impact of environmental regulations and environmental pollution on CER. The main conclusions drawn from this analysis were as follows.

First, mandatory CER disclosure policy can significantly decrease environmental pollution. Environmental information disclosure has become the main means of environmental governance and environmental supervision in many developed countries. However, can China control environmental pollution through environmental information disclosure policies? The conclusion of this paper proves that environmental information disclosure has achieved good results in China. Therefore, environmental information disclosure should be promoted and used as an important policy tool for environmental protection and pollution prevention as soon as possible.

Second, an inverted U-shaped relationship exists between environmental regulations and environmental pollution. When the level of environmental regulation is low, some opportunistic firms will avoid environmental supervision as much as possible, and environmental regulation cannot effectively reduce environmental pollution. Only when environmental regulation reaches a certain level, environmental regulations can help reduce environmental pollution.

Third, air pollution has a positive impact on CER. Air pollution is the most easily perceptible pollution in our lives, and the increase in air pollution will prompt all sectors of society to pay attention to pollution problems. Firms are not only the main producers of environmental pollution but also the main undertakers of environmental protection. Therefore, increased air pollution will promote more environmentally responsible behaviors of firms.

Fourth, an inverted U-shaped relationship exists between environmental regulations and CER. When the level of environmental regulation is appropriate, environmental regulation has a significant role in promoting CER. When the environmental regulation reaches a certain level, too strict environmental regulation will make the environmental cost of the firm too high and affect the enthusiasm of the firm to carry out environmental responsibility activities. Therefore, environmental regulations cannot be too strict or too loose. It is necessary to find a balance between environmental regulations affecting environmental pollution and CER so that they can effectively reduce environmental pollution and increase the enthusiasm of firms to carry out environmental responsibility activities.

This study examined the relationship between environmental pollution, environmental regulations and CER; however, our sample can be considered as a limitation in that it is restricted to emerging countries; future research could explore whether their relationship is different in other countries. Furthermore, although we found that environmental regulations have an inverted U-shaped impact on environmental pollution and CER, this paper has not yet determined what level of environmental regulation is appropriate, which will also be our future research direction.

## Figures and Tables

**Table 1:** Geographical distribution of sample firms.

Eastern		Central		Western	
Province	Firms	Province	Firms	Province	Firms
Beijing	78	Henan	19	Gansu	5
Fujian	42	Heilongjiang	6	Guangxi	7
Guangdong	64	Hubei	17	Guizhou	7
Hainan	7	Hunan	8	Ningxia	3
Hebei	10	Jilin	8	Qinghai	4
Jiangsu	33	Jiangxi	9	Shaanxi	8
Liaoning	17	Inner Mongolia	7	Sichuan	16
Shandong	33	Shanxi	12	Xinjiang	9
Shanghai	70			Yunnan	15
Tianjin	18				
Zhejiang	46				

**Table 2:** Corporate Environmental responsibility measurement.

Dimensions	Indicator	Description	Data Sources
legal consciousness	Global Reporting Initiative Sustainability Reporting Guidelines	If the firm refers to the guidelines, it gets 1 point, otherwise 0 points.	CSMAR
	disclose the environment and sustainable development information	If the firm discloses the environment and sustainable development information, it gets 1 point, otherwise 0 points.	CSMAR
	environmental penalties	If the firm has been punished in terms of the environment, it gets 0 points, otherwise 1 point.	CNRDS
Social evaluation	environmental commendation	If the firm has received an environmental commendation or other positive reviews, it gets 1 point, otherwise 0 points.	CNRDS
	environmental advantages	If the firm has other advantages in the environment, it gets 1 point, otherwise 0 points.	CNRDS
Eco-friendly production	circular economy	If the firm uses renewable energy or adopts circular economy policies and measures, it gets 1 point, otherwise 0 points.	CNRDS
	green production	If the firm adopts policies, measures or technologies to reduce waste water, waste residual, waste gas and greenhouse gas emissions, it gets 1 point, otherwise 0 points.	CNRDS
Low-carbon technology	save energy	If the firm has policy measures or technologies to save energy, it gets 1 point, otherwise 0 points.	CNRDS
	environmentally friendly technologies	If the firm has developed or applied innovative products, equipment or technologies that are more beneficial to the environment, it gets 1 point, otherwise 0 points.	CNRDS
Green management	third-party verification	If the relevant report disclosed by the firm has been verified by a third-party agency, it gets 1 point, otherwise 0 points.	CSMAR
	idea or vision of being responsible for the environment	If the firm has ideas, visions or values that are responsible for the economy, society, and the environment, it gets 1 point, otherwise 0 points.	CNRDS
	ISO 14001 certification	If the firm’s environmental management system is ISO 14001 certified, it gets 1 point, otherwise 0 points.	CNRDS
	green office	If the firm has a green office policy or measure, it gets 1 point, otherwise 0 points.	CNRDS

**Table 3:** Description of variables.

Variables	Description	Samples	Mean	SD	Min	Max
CER	Corporate environmental responsibility	240	4.3682	1.2047	1.0000	7.2179
SO_2_	Sulfur dioxide emissions per square kilometer	240	5.1079	6.2065	0.1622	46.9973
SMOKE	Industrial fumes emissions per square kilometer	240	2.7535	2.5877	0.1639	20.7308
WATER	Industrial effluents emissions per square kilometer	240	0.7642	1.2633	0.0102	7.4036
ER	Environmental regulation	240	0.1946	0.1149	0.0190	0.6516
SIZE	The natural logarithm of total assets	240	1.8545	0.2944	0.8915	2.6405
DEBT	The total debt divided by total assets	240	0.5469	0.0750	0.3995	0.8983
ROA	Return On Assets	240	0.0513	0.2464	−1.0343	3.3186
GDP	Gross domestic product growth	240	10.9358	2.6902	3.0000	17.8200
IND	Industrial added value growth	240	13.0946	5.6806	−4.8000	24.5000
CITY	Urban population divided by total population	240	53.5636	13.4413	29.1100	89.6000

**Table 4:** Panel unit root test.

Variables	LLC	Fisher-ADF
CER	−12.2506 (0.000)	112.6227 (0.000)
SO_2_	−0.2161 (0.4144)	17.5963 (1.0000)
D.SO_2_	−9.6727 (0.0000)	900.0495 (0.0000)
SMOKE	−9.3308 (0.000)	144.2865 (0.000)
WATER	−24.6941 (0.000)	217.1821 (0.000)
ER	−10.0863 (0.000)	157.8388 (0.000)
SIZE	−7.4977 (0.000)	183.0231 (0.000)
DEBT	−9.0662 (0.000)	223.4122 (0.000)
ROA	−15.2217 (0.000)	401.2530 (0.000)
GDP	−30.8663 (0.000)	747.5639 (0.000)
IND	−57.1714 (0.000)	1044.8240 (0.000)
CITY	−10.0863 (0.000)	484.0026 (0.000)

Note: *p*-statistics are shown in parentheses.

**Table 5:** Regression results of environmental regulation and CER on environmental pollution.

Variable	SO_2_	SMOKE	WATER
DCER	−12.6988 *** (1.407)	−0.8391 *** (0.2044)	−0.0865 *** (0.0288)
ER	16.0586 * (8.5663)	4.3355 (3.8496)	1.3563 ** (0.5424)
ER2	−23.1982 * (12.4658)	−3.8518 (5.6494)	−1.8337 ** (0.796)
GDP	0.1654 (0.1402)	−0.0922 (0.0573)	0.0231 *** (0.0081)
IND	−0.0907 (0.0699)	−0.0089 (0.0293)	−0.0124 *** (0.0041)
CITY	0.7713 *** (0.0946)	−0.0726 *** (0.0271)	−0.0106 *** (0.0038)
cons	−30.5562 *** (4.3522)	7.9575 *** (1.4187)	1.1576 *** (0.1999)
R2	0.3175	0.1588	0.1354

Note: *t*-statistics are shown in parentheses below the estimated coefficients. “*”, “**” and “***” denote statistical significance at the 10%, 5% and 1% levels, respectively.

**Table 6:** Regression results of environmental regulation and environmental pollution on CER.

Variable	CER		CER		CER	
	FE	SYS-GMM	FE	SYS-GMM	FE	SYS-GMM
L.CER		0.2873 *** (0.0305)		0.303 *** (0.0427)		0.2501 *** (0.0414)
SO_2_	0.0994 *** (0.0562)	0.1467 *** (0.0236)				
SMOKE			0.0823 ** (0.0386)	0.0619 ** (0.0283)		
WATER					0.5034 (0.3297)	−0.0401 (0.2464)
ER	8.3568 *** (2.9672)	7.1495 *** (1.7747)	8.0598 *** (2.8017)	4.9513 ** (2.2136)	7.5785 *** (2.8218)	4.8206 ** (2.1745)
ER2	−10.1359 ** (4.2189)	−7.5493 *** (2.3524)	−10.2717 ** (4.1053)	−4.7815 (3.2558)	−9.0843 ** (4.1333)	−4.1274 (2.9145)
SIZE	2.8045 *** (0.4329)	1.5053 *** (0.2174)	2.9881 *** (0.3775)	1.4376 *** (0.2573)	3.069 *** (0.3765)	1.8181 *** (0.3549)
DEBT	−2.6864 ** (1.0642)	1.3621 *** (0.4249)	−2.1863 ** (0.9357)	2.1683 *** (0.7551)	−2.4494 ** (0.9596)	1.3487 (0.8549)
ROA	−0.0746 (0.1724)	−0.1102 (0.1607)	−0.0679 (0.1746)	0.2352 (0.2669)	−0.0688 (0.1756)	−0.0052 (0.1741)
GDP	−0.1442 ** (0.0572)	−0.2688 *** (0.0292)	−0.0832 * (0.0497)	−0.3139 *** (0.0284)	−0.0952 * (0.0501)	−0.2815 *** (0.025)
IND	0.0642 ** (0.0263)	0.1026 *** (0.0135)	0.0505 ** (0.0227)	0.1298 *** (0.0088)	0.054 ** (0.023)	0.1169 *** (0.007)
CITY	0.0549 ** (0.0272)	0.007 (0.0083)	0.0704 *** (0.0244)	0.0025 (0.012)	0.072 *** (0.025)	0.004 (0.0171)
cons	8.3568 * (2.9672)		−4.7694 *** (1.2433)		−4.8972 *** (1.2942)	
R2	0.6235		0.6816		0.6781	
sargan		0.5568		0.4636		0.3189
Ar(1)		0.0007		0.0016		0.0018
Ar(2)		0.3338		0.2493		0.2386

Note: *t*-statistics are shown in parentheses below the estimated coefficients. “*”, “**” and “***” denote statistical significance at the 10%, 5% and 1% levels, respectively.

## Data Availability

The data presented in this study are available on request from the corresponding author.
